# Symptoms associated with an abnormal echocardiogram in elderly primary care hypertension patients

**DOI:** 10.1007/s12471-014-0543-y

**Published:** 2014-04-04

**Authors:** L. Ringoir, J. W. Widdershoven, S. S. Pedersen, J. M. Keyzer, V. J. Pop

**Affiliations:** 1 Department of Medical and Clinical Psychology, Center of Research on Psychology in Somatic Diseases (CoRPS), Tilburg University, PO Box: 90153, 5000 LE Tilburg, the Netherlands; 2 Department of Cardiology, TweeSteden Hospital, Dr. Deelenlaan 5, 5042 AD Tilburg, the Netherlands; 3 Department of Cardiology, Thoraxcenter, Erasmus Medical Center, Rotterdam, the Netherlands; 4 Department of Cardiology, Odense University Hospital, Odense, Denmark; 5 Institute of Psychology, University of Southern Denmark, Odense, Denmark; 6 PoZoB, Veldhoven, the Netherlands

**Keywords:** Primary health care, Hypertension, Heart failure, Echocardiography

## Abstract

**Background:**

The prevalence and diagnostic value of heart failure symptoms in elderly primary care patients with hypertension is unknown.

**Aim:**

To assess the prevalence, sensitivity, specificity, positive and negative predictive value of symptoms in association with an abnormal echocardiogram.

**Design and setting:**

Cross-sectional screening study in five general practices in the south-east of the Netherlands.

**Method:**

Between June 2010 and January 2013, 591 primary care hypertension patients aged between 60 and 85 years were included, without known heart failure and not treated by a cardiologist. All patients underwent an echocardiogram and a structured interview including assessment of heart failure symptoms: shortness of breath, fatigue, oedema, cold extremities, and restless sleep.

**Results and conclusion:**

Restless sleep was reported by 25 %, cold extremities by 23 %, fatigue by 19 %, shortness of breath by 17 %, and oedema by 13 %. Oedema was the only symptom significantly associated with an abnormal echocardiogram (positive predictive value was 45 %, sensitivity 20 %, and specificity 90 %, OR 2.12; 95 % CI = 1.23–3.64), apart from higher age (OR 1.06; 95 % CI = 1.03–1.09), previous myocardial infarction (OR 3.00; 95 % CI = 1.28–7.03), and a systolic blood pressure of >160 mmHg (OR 1.62; 95 % CI = 1.08–2.41). Screening with echocardiography might be considered in patients with oedema.

## Introduction

Hypertension is highly prevalent, with the Framingham study showing a lifetime risk for developing hypertension of 90 % in people with a normal blood pressure at age 55 [1]. A high blood pressure can result in coronary artery disease and changes in ventricular function and structure [2], and is a major risk factor for incident heart failure [3], with a 5-year survival rate of 35 % according to previous research [4]. Early diagnosis and treatment of heart failure are crucial for reducing heart failure related morbidity and mortality [5–7]. General practitioners (GPs) are most frequently involved in the diagnosis of heart failure [8]. More severe hypertension in primary care patients is associated with a higher risk of cardiovascular events [9], and lowering blood pressure can reduce risk of major cardiovascular events [10]. However, less than 50 % of patients above the age of 65 are properly managed [11–13]. Therefore, elderly hypertension patients, especially those with less than optimal blood pressure, are at high risk for heart failure.

Several important patient-reported symptoms of heart failure are oedema, dyspnoea, and fatigue [14–16]. Because most patients with possible heart failure will present first in primary care [16], it is important for a GP or practice nurse (PN) to identify symptoms predictive of this condition. However, most studies on symptoms used for assessment of heart failure have focused on hospital populations [8] and/or on patients who presented with dyspnoea [17, 18], or had suspected heart failure [19]. Furthermore, the majority of studies evaluated symptoms only in association with left ventricular systolic dysfunction and did not take other relevant abnormalities such as diastolic function into account [5, 8]. Left ventricular hypertrophy (LVH), an enlarged left atrium, valvular heart disease, and wall motion abnormalities can also be major causes of heart failure and are related to coronary artery disease [20–22].

Echocardiography is seen as the gold standard for the diagnosis or confirmation of cardiac dysfunction associated with heart failure [5] and can be used to detect cardiac abnormalities [2]. Although the diagnostic value of symptoms and signs of heart failure has been the topic of debate [17], attention to symptoms can still be of relevance in patients at risk for heart failure [5].

Until now it is unclear in elderly primary care patients with hypertension which heart failure symptoms may be associated with an abnormal echocardiogram. The objectives of the current study were to examine (1) the prevalence of symptoms which are in general related to cardiac dysfunction reflected by an abnormal echocardiogram, (2) the sensitivity, specificity, positive, and negative predictive value of symptoms of an abnormal echocardiogram, and (3) the relation between the symptoms and an abnormal echocardiogram, after adjustment for several confounders.

## Methods

### Study design and patient population

Between June 2010 and January 2013, primary care patients aged between 60 and 85 years, with an International Classification of Primary Care for hypertension (K86 or K87) as captured from their medical record, were recruited from five general practices affiliated with the primary care organisation PoZoB (a primary care organisation of approximately 200 GPs, located in the south of the Netherlands) for this cross-sectional study. Exclusion criteria were: previous diagnosis of heart failure, current treatment by a cardiologist, history of severe psychiatric illness other than mood/anxiety disorders and/or cognitive impairments (e.g. dementia), terminal cancer, and insufficient knowledge of the Dutch language. This study was approved by the medical ethics committee of the Elisabeth Hospital in Tilburg, the Netherlands.

### Study procedure and data collection

Eligible patients received written information about the study by mail and were asked to sign an informed consent form. Patients were contacted by phone, and in case of informed consent an appointment for an interview at their local GP office was scheduled. This interview consisted of a structured interview of 1 h by a trained nurse. At the end of the intake, an appointment for the echocardiogram was scheduled which also took place at the local GP office.

### Measurements

#### Demographic and clinical variables

Information on demographic and clinical variables was obtained from purpose-designed questions in the interview and included gender, age, marital status, and educational level. Furthermore, blood pressure was measured twice and the average blood pressure was used to dichotomise systolic blood pressure (>160 mmHg was cut-off). Height and weight were measured in order to calculate the body mass index (BMI). Information on clinical variables retrieved from the patients’ medical records included myocardial infarction (MI) and peripheral arterial disease.

#### Assessment of symptoms

Information on heart failure symptoms was obtained during the interview. Structured and standardised questions were used to assess heart failure symptoms. Symptoms were dichotomised (yes/no) and defined as: shortness of breath during moderate exertion (e.g. walking), regularly occurring fatigue, oedema of the legs, ankles and/or feet, having cold extremities, and restless sleep, during the past 4 weeks.

#### Assessment of the echocardiogram

The echocardiogram was performed and evaluated by an experienced echocardiographer of the local primary care laboratory located in Eindhoven, the Netherlands. All the echocardiograms were reviewed by a cardiologist specialised in echocardiography, who indicated if the echocardiogram was abnormal according to a protocol suitable for primary care, based on current guidelines [23, 24]. The definition of an abnormal echocardiogram is shown in Table 1.

**Table 1:** Prevalence of abnormal echocardiogram parameters in 591 elderly primary care hypertension patients

	Total (*n* = 591)
Abnormalities on the echocardiogram	
LAVI >29 ml/m^2^	60 (10 %)
LVEF <55 %	51 (9 %)
Valvular abnormalities	48 (8 %)
LVH septal and posterior wall thickness of ≥13 mm (moderate or severe)	36 (6 %)
Diastolic dysfunction (E/A ratio of <1 and DTs of >200 ms, and presence of LVH in case of grade I diastolic dysfunction)	35 (6 %)
Wall motion abnormalities (hypokinesia, akinesia and dyskinesia)	26 (4 %)
RVH; subcostal wall thickness of ≥6 mm (mild, moderate or severe)	15 (3 %)
Total ≥1 abnormality	175 (30 %)
Total ≥2 abnormality	74 (13 %)

*LAVI* left atrial volume index, *LVEF* left ventricular ejection fraction, *LVH* left ventricular hypertrophy, *RVH* right ventricular hypertrophy, *DT* deceleration time

### Statistical analyses

Statistical analyses were performed using the IBM Statistical Package for the Social Sciences version 18.0. Differences in baseline characteristics were compared between normal and abnormal echocardiograms and assessed with Chi-square tests or Student’s/Welch’s T-tests when appropriate. For each symptom and clinical variable, the sensitivity, specificity, positive predictive value, and negative predictive value with 95 % confidence intervals were calculated.

Adjusted odds ratios were calculated using multiple logistic regression, with an abnormal echocardiogram (taking all cardiac abnormalities together) as dependent variable. A possible relation between symptoms and an abnormal echocardiogram were adjusted for age, gender, education, having a partner, previous MI, peripheral arterial disease, and elevated systolic blood pressure of >160 mmHg.

## Results

### Patient recruitment

From 6 different GP practices, 913 eligible patients with hypertension were approached and 619 (68 %) patients agreed to participate. Of these participants, 2 were excluded post-hoc because they met the exclusion criterion of being treated by a cardiologist, 5 were excluded post-hoc because the echocardiogram was not of sufficient quality and 16 patients did not have an echocardiogram, of which 3 were admitted to the hospital, 1 died, and 12 did not show up. Patients with congenital disease and/or hypertrophic cardiomyopathy were excluded from analyses (*n* = 5), leaving a total of 591 (65 %) patients for analysis.

### Baseline characteristics

Tables 2 shows the baseline characteristics. The mean age of the study patients was 70 ± 6.5 years and 44 % were male (*n* = 262). Patients with an abnormal echocardiogram were significantly older, less often had a partner, were more likely to have had a previous MI, and had a higher systolic blood pressure as compared with the patients with a normal echocardiogram. Abnormalities on the echocardiogram were present in 30 % of the study sample (Table 1).

**Table 2:** Baseline characteristics of 591 elderly primary care hypertension patients

Characteristic	Total, *N* = 591	Normal echocardiogram, *N* = 416	Abnormal echocardiogram, *N* = 175	*P*-value
Demographics				
Male	262 (44 %)	180 (43 %)	82 (47 %)	0.423
Female	329 (56 %)	236 (57 %)	93 (53 %)	0.423
Age, mean (SD)	69.9 (6.5)	69.1 (6.1)	72.0 (7.0)	<0.001
Low education	76 (13 %)	55 (13 %)	21 (12 %)	0.686
Having a partner	443 (75 %)	324 (78 %)	119 (68 %)	0.011
Lifestyle				
Current smoker	77 (13 %)	61 (15 %)	16 (9 %)	0.069
Regular alcohol use (≥2 glasses per day)	188 (32 %)	133 (32 %)	55 (31 %)	0.897
Clinical characteristics and risk factors				
Previous myocardial infarction	27 (5 %)	11 (3 %)	16 (9 %)	0.001
Peripheral artery disease	23 (4 %)	12 (3 %)	11 (6 %)	0.051
TIA/stroke	52 (9 %)	33 (8 %)	19 (11 %)	0.252
Diabetes type 2	67 (11 %)	45 (11 %)	22 (13 %)	0.539
GFR <60 (*n* = 575)	102 (18 %)	72 (17 %)	30 (17 %)	0.962
Mean SBP (mmHg), mean (SD) (*N* = 587)	150 (19.6)	147 (18.0)	155 (22.2)	<0.001
SBP >160 mmHg (*N* = 587)	168 (29 %)	104 (25 %)	64 (37 %)	0.005
Mean DBP (mmHg), mean (SD) (*N* = 587)	82.2 (10.5)	82.0 (10.3)	82.8 (10.9)	0.351
DBP >90 mmHg (*N* = 587)	120 (20 %)	83 (20 %)	37 (21 %)	0.784
BMI (kg/m2), mean (SD)	28.0 (4.5)	28.1 (4.6)	27.7 (4.1)	0.356
Antihypertensive medication				
Diuretics	308 (52 %)	220 (53 %)	88 (50 %)	0.564
Angiotensin receptor blockers	178 (30 %)	137 (33 %)	41 (23 %)	0.021
Angiotensin II receptor antagonists	229 (39 %)	160 (39 %)	69 (39 %)	0.826
Beta-blockers	197 (33 %)	118 (28 %)	79 (45 %)	<0.001
Calcium antagonists	111 (19 %)	66 (16 %)	45 (26 %)	0.005

*TIA* transient ischaemic attack, *BMI* body mass index, *GFR* glomerular filtration rate, *SBP* systolic blood pressure, *DBP* diastolic blood

### Heart failure symptoms and abnormal echocardiogram

Restless sleep was the most prevalent symptom (25 %, *n* = 149). The most sensitive was shortness of breath with a sensitivity of 32 %. The most specific symptom was oedema of the ankles, feet, or legs, with a high specificity of 90 %. Oedema also had the highest positive predictive value (45 %) and negative predictive value (73 %) for an abnormal echocardiogram (Table 3).

**Table 3:** Sensitivity, specificity, positive predictive value, negative predictive value of symptoms associated with an abnormal echocardiogram (*N* = 175/591, 30 %)

	*N* (%)	Sensitivity	95 % CI	Specificity	95 % CI	PPV	95 % CI	NPV	95 % CI
Shortness of breath	102 (17 %)	32.3 %	14.3–20.6 %	71.0 %	66.6–74.9 %	29.6 %	26.0–33.5 %	70.4 %	66.5–74.0 %
Fatigue	110 (19 %)	20.6 %	15.0–27.5 %	82.2 %	78.1–85.7 %	32.7 %	24.3–42.4 %	71.1 %	66.8–75.1 %
Oedema of ankles, feet, legs	78 (13 %)	20.0 %	14.5–26.9 %	89.7 %	86.2–92.3 %	44.9 %	33.7–56.5 %	72.7 %	68.6–76.4 %
Cold extremities	137 (23 %)	28.0 %	21.6–35.4 %	78.8 %	74.5–82.6 %	35.7 %	27.9–44.4 %	72.2 %	67.8–76.3 %
Restless sleep	149 (25 %)	22.9 %	17.0–29.9 %	73.8 %	69.2–77.9 %	26.8 %	20.0–34.8 %	69.5 %	64.9–73.7 %

*NPV* negative predictive value, *PPV* positive predictive value

In adjusted analysis (Table 4), oedema was significantly and independently related to an abnormal echocardiogram adjusted for age, sex, having a partner, the co-existence of type 2 diabetes or peripheral arterial disease, systolic blood pressure >160 mmHg. The other symptoms were not significantly associated with an abnormal echocardiogram in adjusted analysis.

**Table 4:** Correlates of abnormal echocardiogram in elderly primary care hypertension patients (adjusted analysis; *N* = 587)

	Odds ratio (95 % CI)	*P*-value
Unadjusted analysis		
Demographics		
Female gender	0.87 (0.61–1.23)	0.423
Age	1.07 (1.04–1.10)	<0.001
Having a partner	0.60 (0.41–0.89)	0.012
Clinical history		
Previous myocardial infarction	3.71 (1.68–8.16)	0.001
Peripheral arterial disease	2.26 (0.98–5.22)	0.057
SBP >160 mmHg	1.70 (1.16–2.49)	0.006
Symptoms		
Shortness of breath	1.17 (0.74–1.85)	0.505
Fatigue	1.20 (0.77–1.87)	0.428
Oedema of lower extremities	2.17 (1.33–3.53)	0.002
Cold extremities	1.45 (0.97–2.17)	0.073
Restless sleep	0.84 (0.55–1.26)	0.393
Adjusted analysis		
Demographics		
Female gender	0.83 (0.56–1.25)	0.378
Age	1.06 (1.03–1.09)	<0.001
Having a partner	0.71 (0.46–1.11)	0.135
Clinical history		
Previous myocardial infarction	3.00(1.28–7.03)	0.011
Peripheral arterial disease	1.44 (0.70–4.37)	0.230
SBP >160 mmHg	1.62 (1.08–2.41)	0.019
Symptoms		
Shortness of breath	0.90 (0.53–1.53)	0.702
Fatigue	0.92 (0.54–1.56)	0.763
Oedema of lower extremities	2.12 (1.23–3.64)	0.007
Cold extremities	1.30 (0.84–2.01)	0.240
Restless sleep	0.95 (0.61–1.50)	0.836

*SBP* systolic blood pressure, *DBP* diastolic blood

## Discussion

The current study shows that regular restless sleep was reported by 25 %, having cold extremities by 23 %, fatigue by 19 %, shortness of breath by 17 %, and oedema of legs, ankles and/or feet by 13 %. An abnormal echocardiogram was present in 30 %. Oedema is an important symptom of cardiac dysfunction as reflected by an abnormal echocardiogram. In adjusted analysis, oedema was the only symptom that was significantly associated with abnormal echocardiogram apart from higher age, previous MI, and a systolic blood pressure of >160 mmHg. The symptoms shortness of breath, fatigue, having cold extremities, and having restless sleep were of limited value in predicting an abnormal echocardiogram.

### Strengths and limitations

This study is among the first to evaluate the value of heart failure symptoms in the diagnosis of cardiac dysfunction in unselected elderly primary care patients with hypertension. Since hypertension is one of the most important risk factors for incident heart failure [3] and elderly hypertension patients represent a large proportion of patients in whom cardiovascular risk management is crucial, the results of this study are very relevant. By using echocardiography, we studied the association between symptoms and cardiac abnormalities and heart failure, including left ventricular dysfunction, diastolic dysfunction, LVH, and valvular dysfunction. A majority of research evaluated the association of symptoms and left ventricular systolic dysfunction only, and did not take other cardiac dysfunctions into account [8].

Some methodological issues need consideration. First, the cross-sectional design of the study does not allow for evaluation of the course of symptoms in relation to cardiac dysfunction. Second, the study population was primarily Caucasian (99 %) limiting generalisability to other populations with more ethnic diversity. Another limitation is that we do not have detailed demographic information of the non-responders. However, the response rate in the current study was almost 70 %. Moreover, the current design was not used to assess prevalence figures but rather to evaluate whether signs and symptoms could be associated with echocardiogram outcome.

### Comparison with existing literature

Previous research showed that the most common symptoms associated with heart failure are oedema, dyspnoea, and fatigue [14, 15]. However, most previous studies looking at the value of symptoms in the diagnosis of heart failure used selected samples of patients who were referred because of symptoms [25]. For example, patients in the study by Davie et al., in which the symptom oedema had no predictive value with low sensitivity and specificity, were referred for diagnosis of heart failure and the large majority were referred because of symptoms (dyspnoea) [19]. In another primary care study in patients with suspected heart failure similar findings were reported; oedema had limited value in the diagnosis of heart failure, and as in the current study no other symptoms were associated with heart failure diagnosis [17]. However, patients who visited for a specialised hypertension consultation were excluded, limiting the possibility to generalise the results to hypertension patients. Furthermore, in early stages of heart failure, milder symptoms do occur, which are generally insensitive in the diagnosis of heart failure [6]. Nevertheless, a diagnostic tool for heart failure in primary care was developed, based on a systematic review. According to this research, patients who present with symptoms of heart failure are most at risk when there is a history of MI, basal crepitations are present, and the patient is male with ankle oedema. The inclusion of oedema in this tool is more in line with our findings; however, this tool was developed for populations of patients with already suspected heart failure with presentation of symptoms [26], and therefore this tool is most likely not suitable for unselected elderly primary care patients with hypertension patients.

Although breathlessness is the most commonly reported symptom in established heart failure in primary care [16], our findings showed that oedema, and not shortness of breath, was significantly and independently associated with cardiac dysfunction as reflected by an abnormal echocardiogram, together with previous MI, higher age, and elevated systolic blood pressure. However, oedema had a low sensitivity making it less useful for ruling out cardiac dysfunction in elderly hypertension patients. The low sensitivity of all symptoms in our study can possibly be explained by the mild severity of cardiac dysfunction, with the majority of patients not reporting any symptoms.

### Implications

In the majority of cases the GP is involved in the initial diagnosis of heart failure [16]. Therefore, attention to cardiac dysfunction predictive of chronic heart failure is important, especially in primary care populations at increased risk. The results of our study show that oedema of the lower limbs is significantly associated with cardiac dysfunction on an echocardiogram in elderly hypertension patients, it has modest positive predictive value, but adequate specificity. Although the value of symptoms is limited in the diagnosis of heart failure [17], oedema of the lower limbs could be of diagnostic value. The presence of oedema in elderly hypertension patients could alert the GP to initiate further assessment of cardiac function by means of echocardiography. To conclude, it would be recommended for GPs to check for oedema and consider an echocardiogram for further diagnosis of cardiac dysfunction, especially in elderly hypertension patients with a poorly controlled systolic blood pressure and/or a previous MI.
